# Association of some hemostasis and endothelial dysfunction factors with probability of presence of vulnerable atherosclerotic plaques in patients with coronary atherosclerosis

**DOI:** 10.1186/s13104-019-4360-7

**Published:** 2019-06-13

**Authors:** Yu. I. Ragino, E. V. Striukova, I. S. Murashov, Ya. V. Polonskaya, A. M. Volkov, A. V. Kurguzov, A. M. Chernjavskii, E. V. Kashtanova

**Affiliations:** 1 Research Institute of Internal and Preventive Medicine-Branch of the Institute of Cytology and Genetics, Siberian Branch of Russian Academy of Sciences, st.B.Bogatkova 175/1, Novosibirsk, 630089 Russia; 2 The Federal State Budgetary Institution “National Medical Research Center named after Academician E.N. Meshalkin” of the Ministry of Health of the Russian Federation, Rechkunovskaya 15, Novosibirsk, 630055 Russia

**Keywords:** Factors of hemostasis, Factors of endothelial dysfunction, Factor XII, Monocyte chemoattractant protein 1, Stable and vulnerable atherosclerotic plaques in coronary arteries, Relative risk of present of vulnerable atherosclerotic plaques

## Abstract

**Objective:**

The study was dedicated to investigation of some hemostasis and endothelial dysfunction factors association with probability of presence of vulnerable atherosclerotic plaques in coronary arteries in men with atherosclerosis.

**Results:**

The blood levels of factor VII, factor XII and MCP-1 were higher, and concentration of sVCAM-1 lower in men with vulnerable atherosclerotic plaques in the coronary arteries, compared to men who had stable plaques. Have been revealed correlation links between the blood levels of factor II, factor XII, MCP-1 and the presence of vulnerable atherosclerotic plaques in the coronary arteries. Results of logistic regression analysis showed that the relative risk of present of vulnerable atherosclerotic plaques in the coronary arteries is associated with an elevated blood level of factor XII and MCP-1.

## Introduction

Cardiovascular diseases are one of the main causes of mortality in Russia and in the world. The prevalence of ACS also remains extremely high. Initiation of the clinical manifestations of ACS is an erosion or destruction of the endothelium at the site of ulceration/destruction of vulnerable atherosclerotic plaque cover and subsequent thrombus formation and artery occlusion, ischemia and necrosis of the myocardium. Stable plaque is characterized by a thick cover, homogeneous lipid core, the absence of inflammatory changes, and vulnerable by thin cover, or section of thinned cover with focal destruction of the endothelium, the inflammatory cell infiltration, and loose lipid core with areas of necrosis [1, 2].

The endothelial dysfunction and oxidative changes of lipoproteins are known to play an important role at the initial stage of atherosclerotic plaque formation, while at the stage of vulnerable plaque formation the activity of inflammatory and destructive processes is pronounced [1, 3–5].

Dysfunction and destruction of endothelium lead to increased secretion of chemoattractants and adhesion molecules, release of endothelin-1, Willebrand factor in blood, decrease of synthesis and secretion of NO. Disorders of hemostasis are known to accompany almost all stages of atherosclerotic plaque formation. Components of the hemostatic system not only participate in thrombosis of the affected areas of blood vessels, but also can affect the process of formation and progression of atherosclerotic stenosis [5, 6].

In recent years, many studies have been carried out to find and study various pathogenetic biomarkers of coronary atherosclerosis and its complications, especially ACS [7–9].

Purpose of this study was to investigate association of some hemostasis (factor II, factor VII, factor XII, antithrombin III) and endothelial dysfunction (endothelin 1, MCP-1, adhesion molecules sVCAM-1, ADMA, homocysteine, PAI-1) factors/biomarkers with probability of presence of vulnerable atherosclerotic plaques in men with coronary atherosclerosis.

## Main text

### Research methods

The study was conducted in the framework of combined scientific research of Research Institute of Internal and Preventive Medicine-Branch of the Institute of Cytology and Genetics, Siberian Branch of Russian Academy of Sciences and The Federal State Budgetary Institution "National Medical Research Center named after academician E.N. Meshalkin" of the Ministry of Health of the Russian Federation.

The study included 117 men 39–72 years of age with coronary angiographic verified coronary atherosclerosis admitted to the Clinic of the FSBI “National Medical Research Center named academician E.N. Meshalkin” of the Ministry of Health of the Russian Federation on coronary bypass surgery, which during surgery for intraoperative indications was performed endarterectomy from coronary artery/arteries. Exclusion criteria were ACS less than 6 month ago, acute inflammatory conditions, exacerbation of chronic inflammatory diseases, active liver diseases, chronic renal disease, and cancers. Material of endarterectomy containing the intima/media of the artery was transversely divided into fragments, containing atherosclerotic plaque for histological studies. Histological analysis of fragments of the intima/media of the coronary arteries was carried out on a binocular microscope Axiostar Plus (C. Zeiss) with a digital photo output. Stable and vulnerable atherosclerotic plaques differentiated according to the criteria described above [2]. According to the histological conclusion, 54 men (46%) had only stable atherosclerotic plaques in coronary arteries (CA), and 63 men (54%) also had vulnerable plaques in CA along with stable plaques. According to this criterion, all examined patients were divided into two groups.

For biochemical research before coronary artery bypass surgery all the men one-shot after an overnight fast were carried out blood sampling from a vein to obtain plasma and serum. Following hemostasis factors: factor II, factor VII, factor XII, and antithrombin III in the blood plasma were determined by ELISAs method (test system AssayPro). Following endothelial dysfunction factors: endothelin 1 (Biomedica), MCP-1 (Bender Medsystems), sVCAM-1 (Biosource), ADMA (Immunodiagnost), homocysteine (Ahis-Shield), and PAI-1 (Technoclone) were determined by ELISAs method also.

Statistical processing of the results was carried out in the licensed version of SPSS for Windows with the use of correlation, logistic regression and one-way ANOVA analyses using The Dunnet criteria for multiple comparisons.

### The results of the study

According to Table 1, no statistically significant differences were found in the clinical characteristics of the two groups of men (without and with vulnerable atherosclerotic plaques).

**Table 1:** Clinical characteristics of patients

Parameter	Men (n = 54) with stable plaques in the coronary arteries	Men (n = 63) with vulnerable plaques in the coronary arteries	p
Age, years	60.82 ± 7.01	59.71 ± 8.69	0.398
Systolic blood pressure	141.05 ± 16.44	135.68 ± 15.0	0.108
Diastolic blood pressure	86.22 ± 9.51	82.74 ± 9.75	0.920
Pulse rate	67.98 ± 6.11	71.24 ± 7.07	0.280
History of MI	71.4%	76.4%	0.582
History of angina pectoris	100%	100%	0.403
I FC	0	0	
II FC	14.3%	12.7%	
III FC	69.0%	78.2%	
IV FC	16.7%	9.1%	
Essential hypertension	83.3%	87.1%	0.384
Ist	0	3.7%	
IIst	11.9%	5.6%	
IIIst	71.4%	77.8%	
Chronic heart failure	97.6%	100%	0.439
Ist	35.7%	42.6%	
IIst	61.9%	53.6%	
IIIst	0	1.9%	
IVst	0	1.9%	
History of diabetes type 2	11.9%	27.3%	0.064
Overweight (BMI 25–29.9 kg/m^2^)	34.1%	46.3%	0.440
Obesity (BMI ≥ 30 kg/m^2^)	48.8%	42.6%	0.764
Obesity, 1 degree 30–34.9	78.9%	82.6%	
Obesity, 2 degree 35–39.9	21.1%	17.4%	
Obesity, 3 degree ≥ 40	0	0	
Smoking	26.2%	12.7%	0.077
History of hyperlipidemia	76.2%	65.5%	0.252
Multivascular atherosclerotic lesion of coronary arteries (more than two vessels)	92.1%	89.6%	0.689

In patients with vulnerable atherosclerotic plaques in coronary arteries, plasma levels of factor VII and factor XII (Hageman factor) were 1.4 and 1.4 times higher, respectively (p < 0.05), compared with men without vulnerable atherosclerotic plaques in coronary arteries (Table 2). There were no differences between the two groups of men in plasma levels of factor II and antithrombin III.

**Table 2:** Factors of hemostasis and endothelial dysfunction in men with coronary atherosclerosis (M ± σ)

Factors of hemostasis and endothelial dysfunction in the blood	Men (n = 54) with stable plaques in the coronary arteries	Men (n = 63) with vulnerable plaques in the coronary arteries	p
Factor II, µg/ml	252.1 ± 50.9	260.1 ± 58.9	0.474
Factor VII, ng/ml	422.0 ± 147.5	590.1 ± 150.1*	0.016
Factor XII, µg/ml	84.8 ± 58.1	119.9 ± 55.2*	0.010
Antithrombin III, µg/ml	610.9 ± 165.5	600.4 ± 209.6	0.851
Homocystein, µmol/l	18.2 ± 5.2	18.4 ± 8.1	0.626
MCP-1, pg/ml	525.7 ± 188.8	956.8 ± 501.8*	0.015
Endothelin-1, pmol/l	0.8 ± 0.6	1.3 ± 0.6	0.844
ADMA, µmol/l	1.4 ± 0.5	1.4 ± 0.7	0.821
PAI-1, ng/ml	397.5 ± 129.3	399.8 ± 136.5	0.855
sVCAM-1, ng/ml	1017.6 ± 521.8	716.5 ± 345.6*	0.025

*p<0.05

In men with vulnerable atherosclerotic plaques in coronary arteries, serum levels of MCP-1 was 1.8 times higher (p < 0.05) and concentration of sVCAM-1 was 1.4 times lower (p < 0.05) compared with men without vulnerable atherosclerotic plaques in coronary arteries (Table 2). There was no difference between two groups of men in other factors of endothelial dysfunction plasma levels.

Conducted correlation analysis of the studied hemostasis and endothelial dysfunction factors was revealed strong positive correlation links between the blood levels of factor II, factor XII, MCP-1 and the presence of vulnerable atherosclerotic plaques in the coronary arteries (r = 0.275, r = 0.359 and r = 0.397, p < 0.05, respectively).

Multifactorial logistic regression analysis (Table 3).showed that the relative risk of the presence of vulnerable atherosclerotic plaques in the coronary arteries is associated with an increased levels of factor XII (OR = 1.017, 95% CI 1.000–1.049, p = 0.025) and MCP-1 (OR = 1.019, 95% CI 1.002–1.055, p = 0.001) only.

**Table 3:** Logistic regression analysis of the relative risk of unstable atherosclerotic plaques

Factors of hemostasis and endothelial dysfunction	OR	95.0% CI		p
		Lower	Higher	
Factor II	1.002	0.991	1.008	0.856
Factor VII	1.000	1.000	1.004	0.765
Factor XII	1.017	1.000	1.049	0.025
Antithrombin III	1.000	0.994	1.003	0.854
Homocystein	0.799	0.701	1.027	0.353
MCP-1	1.019	1.002	1.055	0.001
Endothelin-1	1.481	0.744	3.004	0.376
ADMA	0.989	0.889	1.025	0.199
PAI-1	0.997	0.981	1.006	0.487
sVCAM-1	0.991	0.868	1.001	0.225

### Discussion

On the 1st stage of our investigation we revealed increase of factor VII and XII levels in patients with vulnerable plaques reflecting the intrinsic and extrinsic coagulation pathway. Extrinsic coagulation pathway is known to be associated with formation of the of tissue factor–circulating factor VII complex participating in such proatherogenic processes as migration and proliferation of vascular smooth muscle cells, inflammation and angiogenesis. The results obtained are in line with other investigations which show association between factor VII activity and cardiovascular diseases [10].

Further analysis of obtained data indicated association of vulnerable plaques presence in coronary arteries with factor XII which take part in two main biological processes—hemocoagulation and formation of kinins, active inflammation mediator [11]. Also factor XII is converging point between processes of inflammation and coagulation [12, 13]. Factor XII activate intrinsic coagulation pathway which starts upon appearing of negative charged surfaces on the surface of endothelium which can be observed in destabilization of the plaque. It could explain revealed associations between Hageman factor and relative risk of vulnerable atherosclerotic plaques presence in coronary arteries. The data obtained are in line with the Kuijpers and co-authors [14] results who have found that the accumulation of factor XII on the external surface of blood clots can regulate the pathological process of thrombosis on the surface of atherosclerotic plaques complicated by rupture. Close affinity between coagulation system and atherosclerosis is proved by investigations that show presence of coagulation proteins in atherosclerotic plaques [15].

Analysis of the data obtained showed association of vulnerable atherosclerotic plaques presence in coronary arteries with increased level of MCP-1. MCP-1 ins not only chemoattractant that provide migration and extravasation of mononuclear cells in focus of inflammation but also inflammation mediator activating in addition resident cells. Oxygenized low-density lipoproteins are known to increase concentration of RNA chemokine MCP-1 which increase leucocytes migration into vascular cell and therefore cause destruction of atherosclerotic plaque surface structures. It could explain revealed association between MCP-1 and relative risk of vulnerable atherosclerotic plaques presence in coronary arteries. Our results are in line with our earlier results of MCP-1 examination immediate in atherosclerotic focuses. MCP-1 level was the highest in vulnerable atherosclerotic plaques [16]. Our results do not contradict the data of other researchers who consider that level of MCP-1 in atherosclerotic plaques, including those with calcinosis, is associated with instability of the plaque and that increased levels of MCP-1 in blood is key for identification of unstable plaques presence in patients with atherosclerosis with a high probability and connected with myocardial infarction development [13, 17, 18].

### Conclusion

Our results indicate that increased blood levels of the Hageman factor and MCP-1 may be a new potential biomarkers of probability of vulnerable atherosclerotic plaques presence in the coronary arteries in men with severe coronary atherosclerosis.

## Limitations

This study is pilot and is limited to a small sample of men with verified coronary atherosclerosis as 97% of patients admitted on coronary bypass surgery were men. The recruitment of women takes a long time and will be analyzed in the future. Further research will allow to determine the range of threshold values, allowing to predict the presence of the atherosclerotic process destabilization.

## Data Availability

The datasets generated and/or analysed during the current study are not publicly available because they are part of a larger dataset which is being reported separately, but are available from the corresponding author on reasonable request.

## Abbreviations

MCP-1: monocytic chemotactic protein-1
sVCAM-1: soluble vascular cell adhesion molecule 1
ACS: acute coronary syndrome
NO: nitric oxide
ADMA: asymmetric dimethylarginine
PAI-1: plasminogen activator inhibitor-1
